# Like, share and follow: The KSSTA and JEO social media

**DOI:** 10.1007/s00167-022-06977-6

**Published:** 2022-04-21

**Authors:** Philipp W. Winkler, Mahmut Enes Kayaalp, Jari Dahmen, Miguel Ángel Ruiz Ibán, Quinten Rikken, Stefano Zaffagnini, Jon Karlsson

**Affiliations:** 1 Department for Orthopaedic Sports Medicine, Klinikum rechts der Isar, Technical University of Munich, Ismaninger Str. 22, 81675 Munich, Germany; 2 Department for Orthopaedics and Traumatology, Dr. Lutfi Kirdar Kartal Training and Research Hospital, Istanbul, Turkey; 3 Department of Orthopaedic Surgery, Amsterdam Movement Sciences, Amsterdam UMC, University of Amsterdam, Meibergdreef 9, Amsterdam, The Netherlands; 4 Academic Center for Evidence Based Sports Medicine (ACES), Amsterdam, The Netherlands; 5 Amsterdam Collaboration for Health and Safety in Sports (ACHSS), International Olympic Committee (IOC) Research Center, Amsterdam UMC, Amsterdam, The Netherlands; 6 Shoulder and Elbow Unit, Hospital Universitario Ramon y Cajal, Madrid, Spain; 7 Facultad de Medicina, Universidad de Alcala de Henares, Alcala de Henares, Madrid, Spain; 8 Facultad de Medicina, Universidad CEU-San Pablo, Madrid, Spain; 9 2° Clinica Ortopedica e Traumatologica, Istituto Ortopedico Rizzoli, IRCCS, University of Bologna, Bologna, Italy; 10 Department for Orthopaedics, Sahlgrenska University Hospital, Institute of Clinical Sciences, Sahlgrenska Academy, Gothenburg University, Gothenburg, Sweden

**Keywords:** Social media, Instagram, Twitter, Facebook, LinkedIn, Altmetric, Metrics, *h*-Index, Journal impact factor

With approximately 4.6 billion active users, social media networks such as Facebook, Instagram, Twitter and LinkedIn are enjoying an increasingly successful career [6]. Even though social media networks are afflicted by a number of criticisms, they can—if used mindfully—definitely enrich one’s private and professional life. Initially conceived as channels to maintain and expand personal social interactions, social media networks have evolved into powerful business tools to attract attention and thus engage followers by disseminating the latest news in a timely manner [10]. This is probably why Kevin Systrom (co-founder of Instagram) once said, *“People are hungry for what's happening right now in the world”*. Translated to the scientific and orthopaedic community, this means that the latest scientific discoveries can be disseminated worldwide by a simple “click” immediately after acceptance by the scientific journal. Only a few years ago, this process of spreading information took several months due to the printing and mailing operations.

**Fig. 1:**
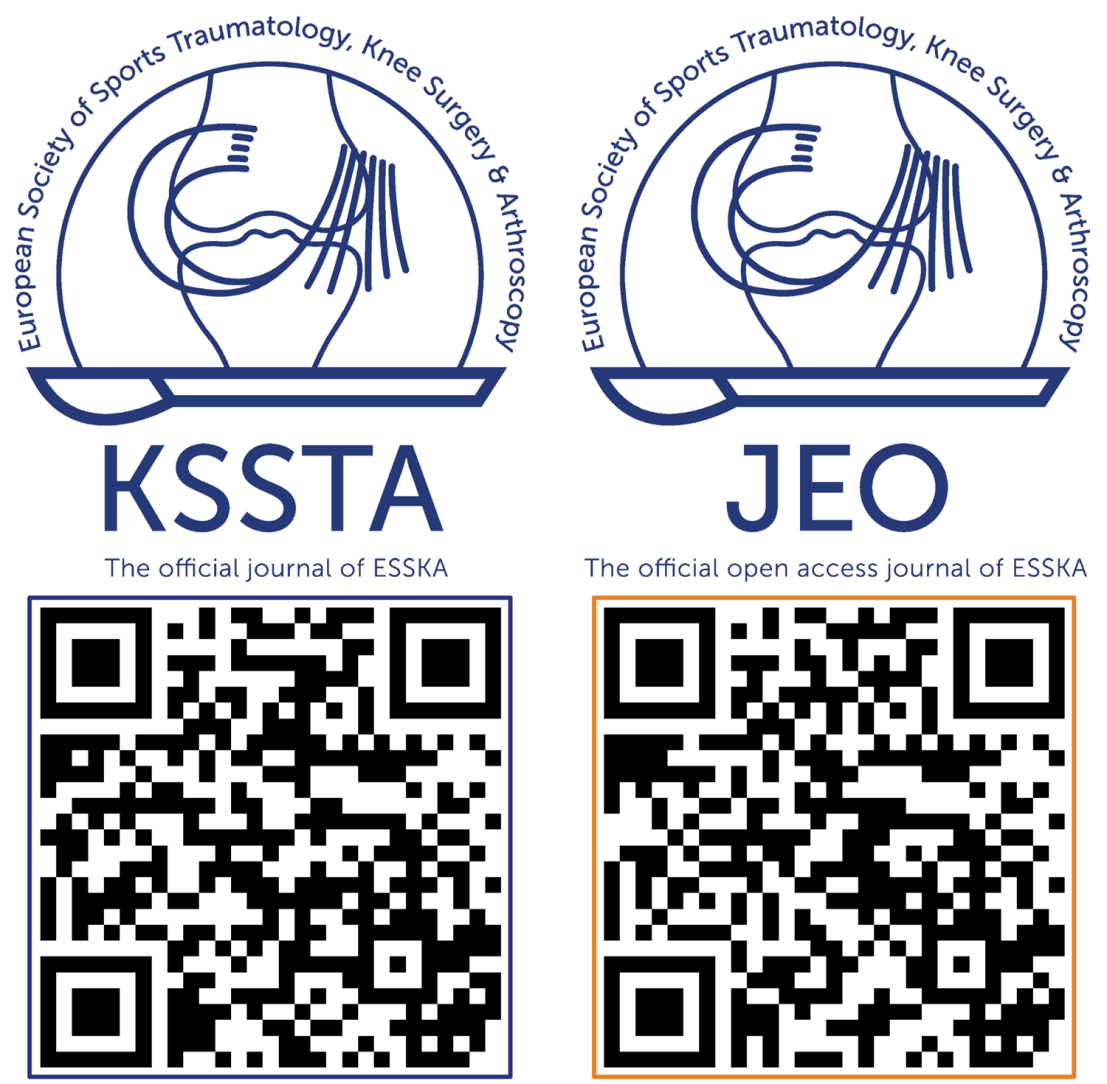
QR code to the most popular social media channel—*Instagram*—from KSSTA and JEO

Given the increasing online presence and profiling of physicians, healthcare institutions, medical companies and scientific journals, their respective digital reputations are becoming increasingly important [11, 15]. Social media are used by physicians and medical companies to advertise in an effort to reach out and engage with patients. Healthcare facilities are increasingly reliant on social media to promote educational and training opportunities to attract qualified students, residents and personnel in general [15]. As a high-ranked scientific journal, *Knee Surgery, Sports Traumatology, Arthroscopy (KSSTA)*, along with others, is also embracing the power of social media networks to disseminate new research findings, encourage interactive and international discussions, engage new readers and thereby enhance the journal’s scientific reputation. Using the QR codes listed here, readers can easily connect to the social media channels of KSSTA and its affiliated journal, *the Journal of Experimental Orthopaedics* (JEO; Fig. 1).

To account for this new level of publication impact, web-based metrics, or *altmetrics*, have emerged as an addition to traditional citation-based bibliometrics such as a journal's journal impact factor (JIF) or an individual's Hirsch index (*h*-index) [2, 5, 9]. Given that the JIF and *h*-index are citation-based metrics, it may take years, perhaps decades, for them to reflect appropriate recognition [2]. In addition, newer means of communication in which scholarly topics are rapidly disseminated and discussed in great depth are not tracked by citation-based metrics. Consequently, *altmetrics* should complement traditional citation-based metrics to add more information about previously invisible discussions and real-time research impact. To better quantify the academic impact of published research, weighted metrics, such as the Altmetric Attention Score (AAS), exist to allow various sources (i.e., social media networks, newspaper, blogs etc.) to be scored differently, reducing the risk of skewed metrics [1]. However, there are also several drawbacks in relation to the AAS, as its calculation disregards some popular platforms, such as Instagram. It is also worth noting that the Twitter weight in the score calculation is four times the weight of Facebook and news and blog sources are six to eight times more valuable than a Twitter mention [4]. Taking account of the unequal popularity of social media platforms across countries, where Twitter has almost 78 million users in the US alone and 18 million in France and Germany together [7], the AAS of an article might end up being geographically related. These concerns make it necessary for readers to be careful while evaluating the AAS of an article and even more so when comparisons are made between the AASs of different articles.

**Fig. 2:**
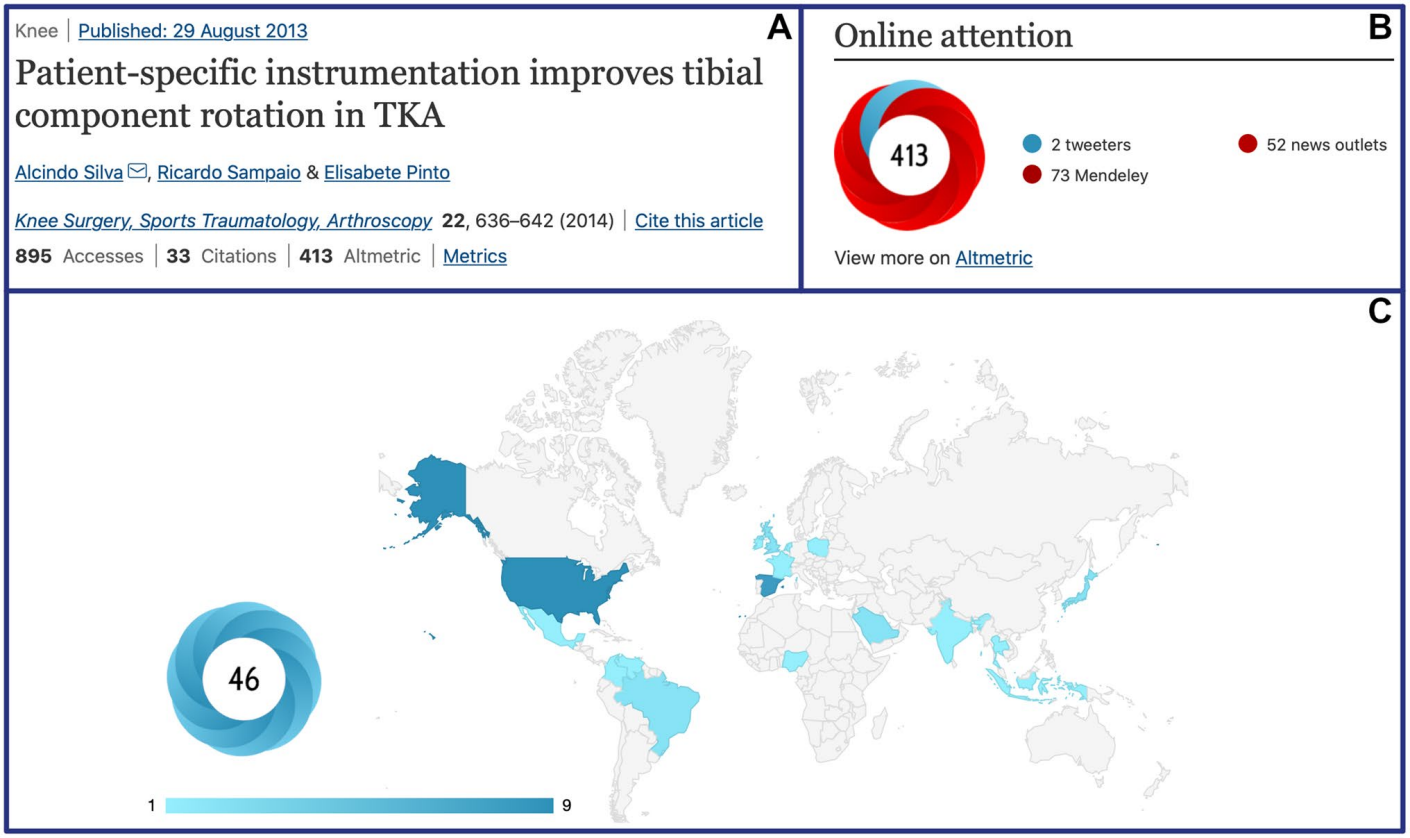
Article metrics provided by KSSTA. **A** KSSTA article with the all-time highest Altmetric Attention Score (AAS) on the publishing website [12]. **B** Details on the article’s online attention, including the Altmetric donut, are displayed after clicking *“Metrics*”. **C** Geographical breakdown of another article’s online attention with an AAS of 46 can be visualised by clicking *“Altmetric”* [13]

Apart from all the controversy, KSSTA and JEO have already embraced *altmetrics* and they can easily be found through the journal’s publishing website (https://www.springer.com/journal/167 and https://jeo-esska.springeropen.com). Once the website has been accessed and the desired article has been selected, the most important metrics can be obtained by simply clicking on *“Metrics”* just below the list of authors. At this point, traditional metrics, such as the number of article accesses and citations based on Web of Science and CrossRef, can also be accessed. In addition, the online attention of the respective article is indicated by the AAS and visualised by the Altmetric donut. The highlighted number in the centre of the donut represents the AAS of the article, while the different colours of the donut reflect different sources of online attention such as Twitter, Facebook, LinkedIn and others. For even more detail, click on *“Altmetric”* for insights into the demographic and geographical online attention of the respective article (Fig. 2).

It is noteworthy that a high level of social media attention (i.e., many likes, shares, or retweets and high engagement rates) for published articles does not necessarily equate with high-quality research. Ultimately, the scholarly quality of research should be critically appraised by each individual scientist. However, a study investigating the association between web-based metrics and citation-based metrics revealed a statistically significant and positive correlation (*r* = 0.32) between the AAS and the citation rate of 496 articles published in five major orthopaedic journals in 2016 [8]. Similar relationships between web-based metrics and citation-based metrics have already been demonstrated in other fields of medical research [3, 14].

**Fig. 3:**
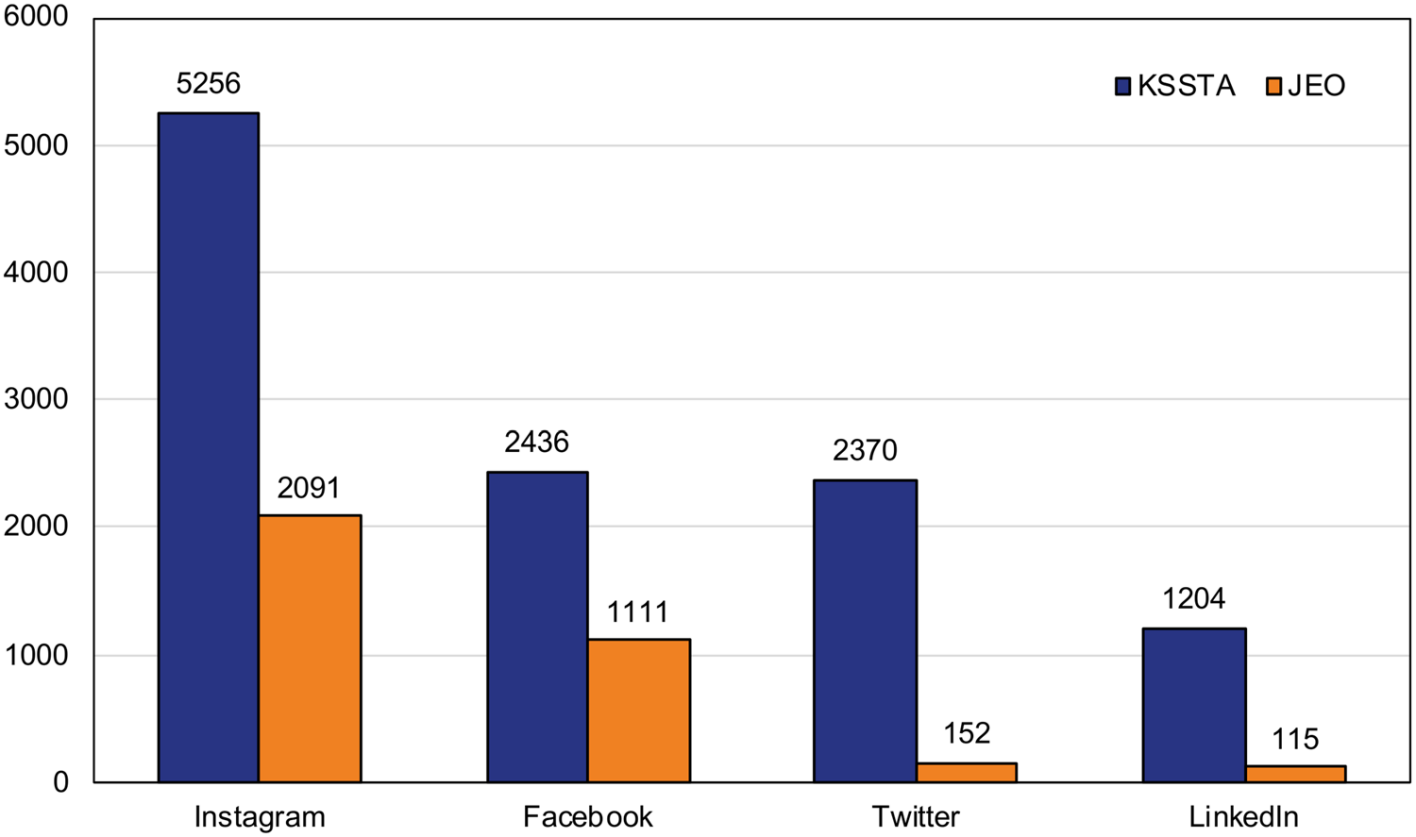
Number of followers on all available social media channels for KSSTA and JEO (retrieved on January 30th, 2022)

Given that high web-based attention is positively associated with and at least weakly predictive of future citation rates [8], social media networks to promote and disseminate research outputs are valuable instruments for KSSTA and JEO to increase their academic impact. Accordingly, KSSTA and JEO have launched social media channels on Facebook, Instagram, Twitter and LinkedIn (Fig. 1). Since the start, almost 400 scientific articles have been posted on the social media platforms that are tracked by more than 5,000 followers, with a constantly increasing number of followers across the different social media channels (Fig. 3).

In order to facilitate the growing online presence, five web editors are currently working for KSSTA (MEK, JD, PWW) and JEO (MARI, QR) respectively. This collaborative board of web editors, supported by the editors-in-chief (JK, SZ), means that it features at least four articles a week on all social media channels, assists the editorial board with online outreach and introduces new tools, such as podcasts and infographics in the future, to constantly improve the visibility of KSSTA and JEO. In addition, the hard work of all the authors can be acknowledged by disseminating their scientific findings. The collaboration between the editorial boards of KSSTA and JEO further enhances the harmonisation of the submission process for both journals, which aims to give authors a greater chance of publishing. Moreover, it allows the web editors to select articles with similar research topics and create combined KSSTA and JEO posts.

Taken together, web-based scholarly discussions are more popular than ever. Social media networks enable scientific journals such as KSSTA and JEO to contribute to these discussions and disseminate new research output globally in a timely manner. Alternative web-based metrics enrich the scientific community by complementing traditional citation-based metrics, thereby collating all the potential impacts of research findings. However, *altmetrics* do not tell the whole story. It is still the responsibility of the individual scientist to evaluate the quality of published research. To enhance a scientist's reputation, underscore important research findings and not least acknowledge the publishing journal, scientists are advised to promote their scholarly achievements digitally. KSSTA and JEO appeal to their social media-active members to share, like and follow posts that pique their individual interests, thereby helping to disseminate the high-quality content of the journals.
